# A strategy for large-scale comparison of evolutionary- and reaction-based classifications of enzyme function

**DOI:** 10.1093/database/baaa034

**Published:** 2020-05-25

**Authors:** Gemma L Holliday, Shoshana D Brown, David Mischel, Benjamin J Polacco, Patricia C Babbitt

**Affiliations:** 1 Department of Bioengineering and Therapeutic Sciences, University of California, San Francisco, San Francisco, 1700 4th Street, CA 94143, USA; 2 Department of Pharmaceutical Chemistry, University of California, San Francisco, San Francisco, 1700 4th Street, CA 94143, USA; 3 Quantitative Biosciences Institute, University of California, San Francisco, San Francisco, 1700 4th Street, CA 94143, USA; 4 Present Address: Medicines Discovery Catapult, Mereside, Alderley Park, Alderley Edge SK10 4TG, UK

**Keywords:** Enzyme classification systems, Evolution- versus reaction-based enzyme classification, Enzyme function, Comparison of enzyme classification systems, Structure–function relationships, Structure–reaction relationships, Functional annotation

## Abstract

Determining the molecular function of enzymes discovered by genome sequencing represents a primary foundation for understanding many aspects of biology. Historically, classification of enzyme reactions has used the enzyme nomenclature system developed to describe the overall reactions performed by biochemically characterized enzymes, irrespective of their associated sequences. In contrast, functional classification and assignment for the millions of protein sequences of unknown function now available is largely done in two computational steps, first by similarity-based assignment of newly obtained sequences to homologous groups, followed by transferring to them the known functions of similar biochemically characterized homologs. Due to the fundamental differences in their etiologies and practice, `how’ these chemistry- and evolution-centric functional classification systems relate to each other has been difficult to explore on a large scale. To investigate this issue in a new way, we integrated two published ontologies that had previously described each of these classification systems independently. The resulting infrastructure was then used to compare the functional assignments obtained from each classification system for the well-studied and functionally diverse enolase superfamily. Mapping these function assignments to protein structure and reaction similarity networks shows a profound and complex disconnect between the homology- and chemistry-based classification systems. This conclusion mirrors previous observations suggesting that except for closely related sequences, facile annotation transfer from small numbers of characterized enzymes to the huge number uncharacterized homologs to which they are related is problematic. Our extension of these comparisons to large enzyme superfamilies in a computationally intelligent manner provides a foundation for new directions in protein function prediction for the huge proportion of sequences of unknown function represented in major databases. Interactive sequence, reaction, substrate and product similarity networks computed for this work for the enolase and two other superfamilies are freely available for download from the Structure Function Linkage Database Archive (http://sfld.rbvi.ucsf.edu).

## Introduction

Historically, examinations of enzyme chemistry and enzyme evolution have been done using separate and distinct approaches to organize, compare and disseminate each type of data. This is because there is no easy way to process relationships between an enzyme’s chemistry and its cognate sequences and structures together, except through small scale and focused studies in which enzyme mechanism can be explicitly associated with the specific sequence and structural features that enable catalysis. While a large compendium of tools and data resources for understanding the relationships between enzyme sequences and their structures (bioinformatics) is well established, the tools and resources to describe the chemical relationships of enzymes (cheminformatics) are less mature, complicating our ability to link the chemical and the protein perspectives together in a computationally useful way.

Although extremely sparse compared to sequence data, experimentally validated reaction annotations are heavily leveraged for large-scale annotation transfer from biochemically characterized enzymes to homologous sequences of unknown function (unknowns; see, for example, (1–3)). The most foundational source for naming experimentally determined enzyme reactions is provided by the Enzyme Commission (EC), which defines catalytic reactions using a hierarchical set of four-digit numbers that run from least to most specific descriptors (4, 5). (Importantly, for this work, the third digit of the EC system designates an overall enzyme reaction, while the fourth digit designates substrate specificity.) In addition to the enzyme nomenclature data, many other resources now provide online access to more in-depth types of information about enzyme chemistry, including overall chemical transformations and functional features, such as kinetic details and mechanisms of reactions. These may also include some sequence features, e.g. active site residues with descriptions of their functions. BRENDA (6), SABIO-RK (7), KEGG (8,9), MetaCyc (10) and the reaction-related information in UniProtKB (11) represent varied examples.

Despite their value, several issues limit the potential of chemistry-centric resources for linking the chemical and evolutionary perspectives. First, while it provides a systematic naming convention for enzyme reactions, the EC classification system remains uninformed by the evolutionary perspective (12). Second, as EC annotations define a single reaction at a time, the EC system lacks a sufficiently sophisticated conceptual infrastructure for linking chemical data to large sequence superfamilies that may represent many different reactions. Further limiting the creation of large-scale resources describing enzyme chemistry, the amount of available mechanistic information remains tiny in comparison to the enormous volumes of uncharacterized sequences. As a result, even as new biological resources continue to emerge that relate enzyme proteins and their reactions (13), the difference in the size of each data type raises challenges for connecting the large sequence space of enzymes with their chemical capabilities.

However, new resources that more effectively include both the chemical and evolutionary perspectives are emerging. These resources capture both small- and large-scale information about enzyme reactions and associate these data to the proteins that enable them (for examples see (10, 14–16)). Related to these types of efforts, support for large-scale functional analysis of enzyme domains described in the CATH database (17) is now used to enhance the information FunTree (18) provides through the addition of similarity measures for enzyme reactions and their associated metabolites (19). In another example, the Structure–Function Linkage Database (SFLD) (20) directly links sequence and structural conservation patterns with their roles in catalysis, providing a foundation for deeper integration of the protein- and chemistry-centric perspectives. Still, for many protein-centric resources, including the SFLD, the associated chemical context has been historically captured only by simple descriptors using static images of overall reactions.

Thus, additional work needs to be done to bridge the conceptual disconnects between the protein- and reaction-centric perspectives and to support direct comparison of the two types of classification systems. To achieve this, a formal way to link the two types of information together in a computationally intelligent manner will be required. Examples of new work aimed at addressing this challenge are emerging including the creation of the Mechanism and Catalytic Site Atlas (M-CSA) (21) and Biochem4j (22). The former represents a merger of the Mechanism, Annotation and Classification in Enzymes (MACiE) (23) database and the Catalytic Site Atlas (CSA) (24). The integrated M-CSA resource connects information about conserved catalytic site residues with sets of homologous proteins with which they can be associated. Using a different type of approach, Biochem4j introduces a graph database framework to connect chemical reaction, enzyme and taxonomic data. Additionally, the advent of resources such as the Rhea resource of biochemical reactions (25) is helping to address the disconnect between the stand-alone EC system and sequence similarity data. Rhea generates chemical entities from the ChEBI ontology (26) and now provides reaction data to UniProt. This collaboration also enables facile programmatic access to reaction data across the large suite of tools and data resources available from EMBL-EBI web services (27).

In this work, we present a new computational strategy designed to enable more informative comparison of enzyme function classification from the perspective of the chemical reactions they catalyze with that of the homologous proteins that enable them. Named MEERCat (Mechanism and Evolution in Enzyme Reaction Catalysis), our strategy links two ontologies previously developed to support the independent classification of enzymes from each perspective. Using the large and well-studied enolase superfamily (28, 29) as a gold standard, this retrospective analysis reveals a substantial disconnect between the two classification systems and raises new concerns for the facile transfer of biochemical function from chemistry-based classifications of biochemically characterized enzymes to their sequence homologs of unknown function.

For this proof-of-concept report, the evolutionary perspective was provided using the SFLD as a data resource platform, as it already incorporated the evolution-based ontology used for this study. Addition of the chemistry-centric perspective was patterned after the ontology developed for the MACiE database. Chemical classification features from MACiE were added to the SFLD to enable us to formally link reactions and ligands to functionally diverse enzyme superfamilies curated by the SFLD.

Functionally diverse enzyme superfamilies (29) are composed of nonredundant sequences found in a multitude of organisms. Each superfamily may contain many thousands of such sequences. For example, the enolase superfamily in the SFLD archive is comprised of over 50 000 sequences while the radical SAM superfamily is comprised of over 100 000 nonredundant sequences (30) (as of 2017 when the test set of data was collected for this project). All of the member sequences in such superfamilies conserve key structural and sequence features associated with a fundamental chemical capability. In the enolase superfamily, all of its varied reactions use a conserved constellation of active site residues to initiate a common partial reaction, abstraction of a proton alpha to a carboxylate, that leads to the formation and stabilization of an enolate anion intermediate (28). Additional reaction steps then enable the different overall reactions of the superfamily.

Functionally diverse superfamilies typically have domain architectures and fold types that nature has repeatedly retooled to catalyze many different enzyme reactions. For example, the enolase superfamily belongs to the (β_8_/α_8_) triosephosphate isomerase (TIM) barrel fold class, a structural scaffold in which key catalytic residues are located around the center of a symmetrical active site `barrel’ where the substrate binds. The high representation of the TIM barrel fold in many enzyme superfamilies has been acknowledged as due in part to the relative ease at which variations in this active site architecture can evolve (31). This and other functionally diverse superfamilies represent useful models for the work described here as their homologous members all have similar structures and some conserved active site features, yet catalyze quite different overall reactions. In such superfamilies, simple annotation transfer of EC number from characterized members to sequences of unknown function is prone to high levels of misannotation (32).

The curated superfamily data and similarity networks resulting from this work are freely available from the SFLD archive (http://sfld.rbvi.ucsf.edu) along with links to documentation, tutorials and other help files. SFLD curation for the enolase and radical S-adenosylmethionine (SAM) superfamilies is also available from the InterPro Resource (33). Although MEERCat lays out a first pass blueprint for knowledge representation and integration of the ontologies for relating protein-centric and chemistry-centric enzyme classification systems, the SFLD itself is no longer being actively developed or maintained. Thus, it will be the task of future work to implement an active resource enabling new comparisons using a MEERCat-like strategy.

## Results and Discussion

### Development of the MEERCat strategy

At its simplest, an enzyme can be described as a protein that performs catalysis. From this chemistry-centric viewpoint, substrates and products can be compared, as can catalytic functions. These can be described in chemical terms for such properties as overall and partial reactions with respect to associated chemical changes (e.g. bonds formed, cleaved and changed in order). From a protein-centric viewpoint, features of proteins can be represented in an evolutionary context in which sequences and structures can be compared among homologous members of a superfamily to identify conserved features likely to be associated with their specific molecular functions. The strategy reported here captures both protein- and chemistry-centric types of information by linking together ontologies representing each. Biological context, a third key context for describing proteins, including enzymes, is captured by many other resources, such as the Gene Ontology. This aspect of enzyme function is beyond the scope of this work and will not be discussed further.

### Linking ontologies describing evolutionary and chemical classification systems for enzymes

The two ontologies used in this study are the Enzyme Structure–Function Ontology (ESFO) (34) and the Enzyme Mechanism Ontology (EMO) (24). The ESFO describes annotation transfer of catalytic function based on sequence similarity. (See http://purl.bioontology.org/ontology/ESFO for a list of terms in the ESFO.) The EMO describes catalytic characteristics of enzyme function in terms of reactions and their associated small molecules. (See http://purl.bioontology.org/ontology/EMO for the full list of the terms relating to residue annotation.)

#### The Enzyme Structure–Function Ontology

The ESFO provides the evolution-based framework specified in the SFLD for classifying sequence and structure variations that have produced the varied contemporary reactions known for many functionally diverse superfamilies (35, 36). It defines sequence–function relationships in the SFLD in terms of an evolutionary hierarchy in which sequences are classified from a top-down viewpoint (functionally diverse superfamily > subgroup > family). Conceptually, the superfamily is the highest level of the hierarchy and includes sequences deemed to be homologous but that can be functionally diverse at all four levels of the EC system (29). Within a superfamily, subgroups are defined as more granular subsets for which the sequences within any particular subgroup are all more similar to each other than they are to the sequences within a different subgroup. Each subgroup is associated with conservation of additional protein features not conserved in all members of the superfamily, with each distinct subgroup likely arising by paralogous descent from the common ancestor. Typically, conserved active site features found in a specific subgroup can be associated with some common functional and structural properties even though their members may represent multiple different overall reactions. (See the original definition of enolase superfamily subgroupings for examples of how they differ in sequence and their associated functional properties (28).) These may include reactions classified as different at least at the third digit of the EC system and sometimes even at the first and second EC digits. Within subgroups, reaction families are defined as proteins that catalyze the same reaction using the same basic mechanism. Of these hierarchical levels, only reaction families are considered monofunctional (at the third level of the EC system).

**Figure 1:**
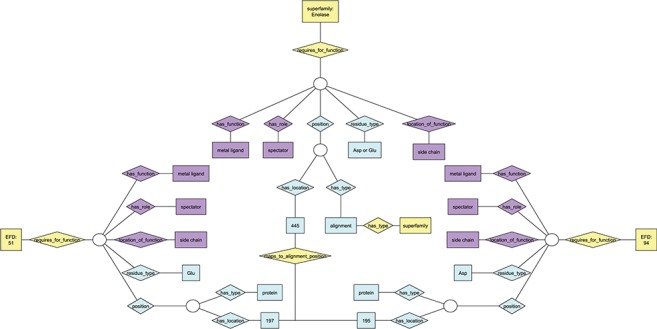
Ontology view of annotation for functional residues. Diamonds represent relationships; rectangles represent terms in the ontologies. Purple nodes, annotation details from EMO; yellow nodes, annotation details from the EFSO; blue nodes, details of specific residue location and type. EFDs shown in this figure are the same as those describing UniProtKB protein Q97U27 (EFD:51, gluconate dehydratase family) and UniProtKB protein P11444 (EFD:94, mandelate racemase family) in Figure S1. Though each of the two EFDs shown has multiple functional residues, only one is shown in the figure due to space constraints. The chosen residue has the same functional role in each EFD (and maps to the same position in the MSAs for the subgroup and superfamily that include both EFDs), though the amino acid type differs (Asp at position 195 for EFD 94, Glu at position 197 for EFD 51).

The ESFO contains an additional formal concept, the enzyme functional domain (EFD). The EFD is defined as the smallest contiguous amino acid sequence (domain) that is required for a specific function to be performed. It formally links the evolutionary context with specific functional properties defined in terms of a specific overall reaction. Figure S1 provides a simple example. Note that the definition of the term `domain’ for the EFD differs from that defined using sequence or structural information alone, e.g. as defined by Pfam (37), in that it represents the complete sequence required for that function to occur. As a result, the EFD may contain multiple structural domains.

Information required for classification of superfamily members into reaction families in the SFLD has typically been obtained *via* manual curation of a `canonical’ family protein with clear experimental evidence for catalysis of a specific overall reaction. Using information derived from the literature or from high-confidence resources such as the SwissProt part of the UniProtKB, key criteria for family membership, e.g. required catalytic residues, are then defined using this protein. These functional residues are stored at the EFD level and this information is propagated upwards at each level of the hierarchy as far as can be determined from their conservation in the associated multiple sequence alignments (MSAs). In most cases, the specific residue type is conserved throughout the hierarchy. However, occasionally, the function a residue performs is conserved while its specific type is not. Using this information, experienced curators then add sequences of unknowns to a reaction family based on their similarities to the defined family profile (38).

#### The Enzyme Mechanism Ontology

The chemistry-centric framework implemented in the SFLD for this work uses the EMO, previously developed for use with the CSA and MACiE databases. The EMO was designed to enable representation and comparisons of chemical characteristics in terms of the reactions and the small molecules on which they work. It has been used to facilitate annotation transfer among other resources, including the UniProtKB. It provides a formal description of functional residues, including the identity and role a residue plays in a specific reaction and its position in the sequence. The annotation captured by the MEERCat strategy describes the complete transformation required to restore an enzyme to its initial state, e.g. a proton shuttle in which a residue acts as both a general base and general acid. This representation may differ from how a specific residue is often described in the literature and in the SFLD, i.e. in a mono-directional manner, only as a general base.

Prior to the work described here, enzyme reactions were provided in the SFLD only as static .gif images and SMILES (simplified molecular-input line-entry system) strings (39). Following the EMO, enhanced annotation of functional residues could be added to the SFLD using the EFD to link the ESFO and EMO. By using these two ontologies together, we could formally incorporate conserved chemical components into the SFLD architecture in a way that enabled the direct comparison of evolution- and reaction-based enzyme classifications. In contrast to the top-down organization of enzyme superfamilies specified by the ESFO, however, the EMO classifies sequence–function relationships starting from a specific protein, such as is used by MACiE. As the EFD includes in its definition the functional (catalytic) residues that are critical for the function of specific enzymes in a reaction family, it is the only level at which an overall chemical transformation can be definitively mapped with the ESFO (Figure 1).

### Formal representation of conserved chemical components

A key design concept of the original SFLD that distinguishes it from other databases that describe relationships only between sequences and/or structures is its inclusion of a conserved chemical feature associated with all members of a superfamily (40). This concept motivated creation of the SFLD to enable explicit analysis of structure–function relationships in functionally diverse enzyme superfamilies. The strategy reported here added to the simple mapping of reaction information in the original SFLD. The formal representation of these chemical relationships that resulted is both computationally tractable for large-scale analysis and richer and more sophisticated than that initially conceptualized in the SFLD design.

**Figure 2:**
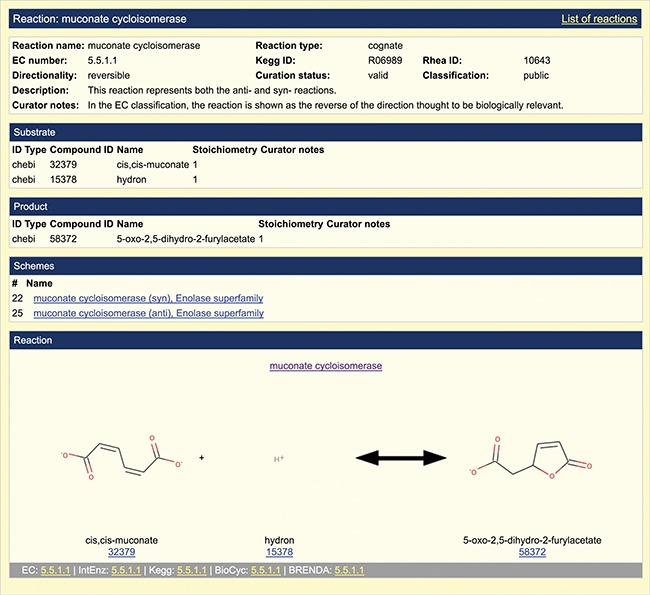
Representation of overall reaction. A screenshot of the default reaction view available in the SFLD archive following incorporation of the EMO into the SFLD resource is shown. The muconate cycloisomerase (EC 5.5.1.1) reaction (enolase superfamily) is shown as an example.

Molecular representation of chemical molecules can be described in many different ways in order to capture reactions in a computer readable format (41). The approach used in this work stores a list of molecule identifiers to capture substrates and products and relate these back to information gathered from external databases, primarily the ChEBI database. (These identifiers were also used in the creation of chemical similarity networks, described in the sections below and in Methods.)

The full schema for reaction representation implemented in the SFLD using our strategy is shown in Figure S2. Details of the annotation relationships linked through the EFD for two members of the enolase superfamily are shown in Figure S3. In some superfamilies, chemically different amino acid residues may be used to perform the same function, e.g. the conserved chemical component may reflect a general chemical strategy, such as stabilization of an oxyanion hole, rather than a specific partial reaction (42).

### Overall reaction information added to the SFLD

**Figure 3:**
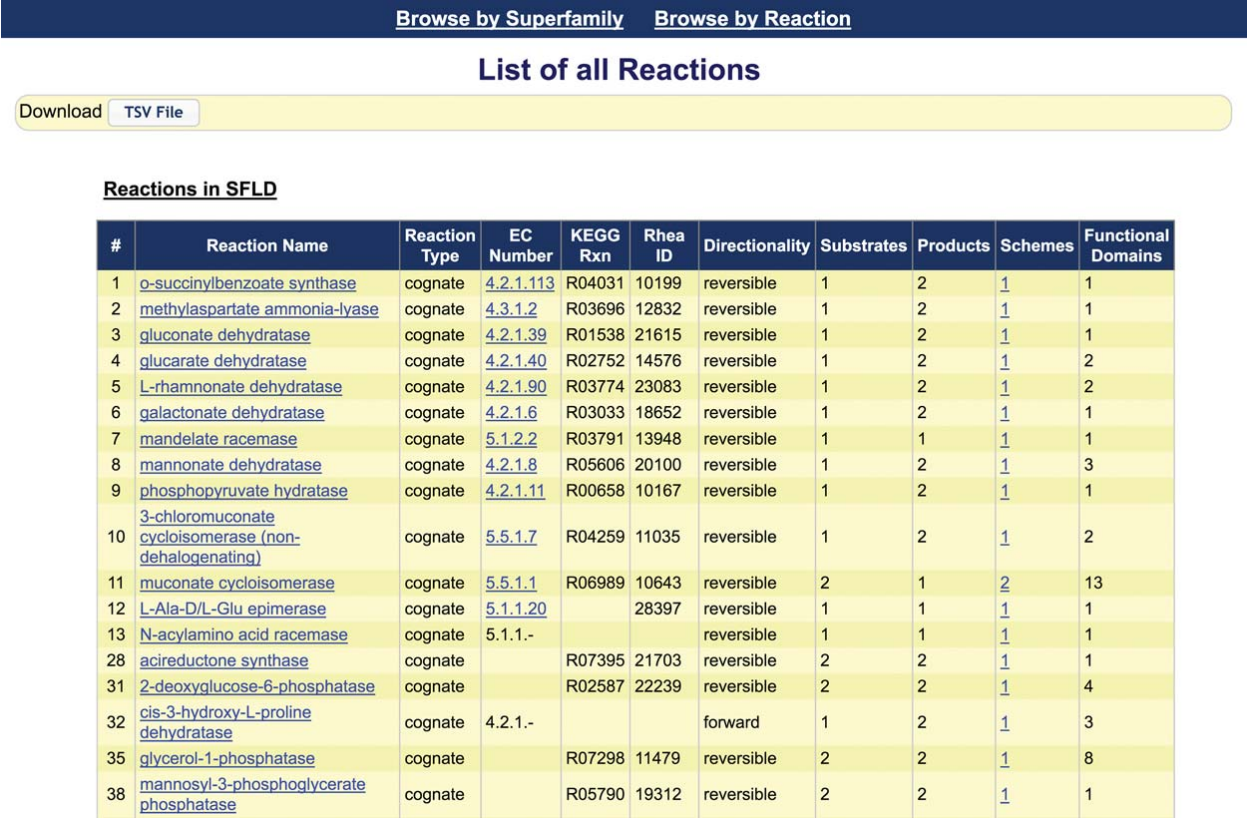
Screenshot of the first 18 reactions listed in the SFLD. The information available from this web page includes the assigned name of each reaction, reaction type, links to the EC number and identifiers for relevant outside resources, directionality (forward, backward and reversible) and counts of the number of substrates, products, reaction schemes and functional domains assigned by curators. (The complete reaction list includes some reactions curated using MEERCat but for which the associated protein-centric data is not curated in the public SFLD archive.) The left-most column represents the number of the unique SFLD reaction identifiers for each reaction in the downloadable TSV file of all the reactions available in the SFLD Archive.

Information about overall chemical transformations (reactions) is represented as a collection of molecules stored as the starting state (substrate(s)) and final state (product(s)) of the transformation. Figure 2 provides an example of new information that was added to the SFLD for this work. The addition of these reaction-centric views produces a much enhanced representation of enzyme structure–function relationships than was previously available. The `Browse by Reaction’ page (Figure 3), available from the menu bar on the home page of the SFLD Archive, allows users to browse curated reactions by their annotation fields.

In the example shown in Figure 2, links to EC number, IntEnz (43), KEGG, BioCyc and BRENDA are provided. The full reaction entry shown uses arrows to depict reaction directionality as reversible, which is the default option, or as forward or backward as appropriate. For reactions in which the directionality is unknown, the arrow is replaced with a question mark. This is all handled with a directionality tag in the reaction annotation. For cases in which a reaction represents more than one mechanism (reaction scheme), each overall reaction is treated as unique. An overall reaction is only annotated once, even if that reaction is seen in multiple reaction schemes (mechanisms). For example, the reaction scheme shown in Figure 2 formally links the reaction to the two EFDs that represent two families that catalyze the same reaction but use different mechanisms (44). Multiple mechanisms for the same reaction can also be exhibited in different superfamilies.

This proof-of-concept study added reactions for only a limited set of superfamilies curated in the SFLD (the enolase, radical SAM and haloacid dehalogenase superfamilies (45)). The listing of available reaction pages that are provided is accessible from the `Browse by Reaction’ link on the menu bar of the SFLD Archive. Figure 3 provides a screen shot of the first 18 of these. The reaction pages added to the SFLD include other new types of information as well. These include reaction name and reaction type. In nearly all cases, this is the biologically relevant cognate reaction, but the reaction could also be annotated as generic, i.e. the substrates and products contain a generic R group. Other information includes EC number, directionality (mostly reversible) and substrate and product counts. Counts are also included for the number of available reaction schemes and functional domains associated with each named reaction. Where a fully defined EC number was available when the test set of reactions was gathered in 2017, links are provided to ExplorEnz. [Note that although a new primary (first digit) class was added to the EC system in 2018, none of those enzymes are included in the enolase superfamily. We have not evaluated whether there are other reactions in the SFLD that would be affected by the addition of Class 7.] The number of overall reactions available for each of these superfamilies can also be found on the superfamily summary page accessible from the `Browse by Superfamily’ link on the SFLD Archive menu bar and from the EFD and family pages.

**Figure 4:**
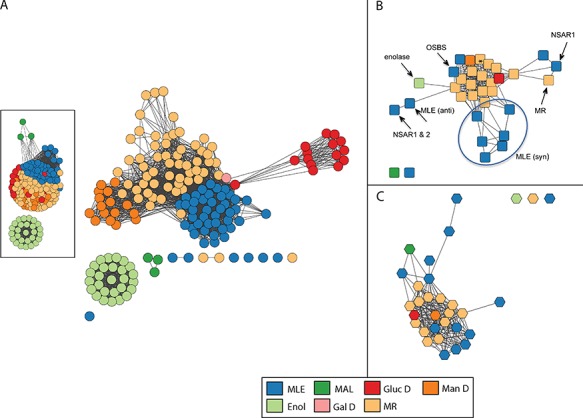
Similarity networks for the enolase superfamily colored by SFLD subgroup. Network visualization uses the organic layout provided by the Cytoscape software (66). In this layout, edges are drawn between nodes if the similarity score is ≥ a statistical significance threshold pertinent to the comparison metric; edge lengths correlate with the degree of connectivity. (A) Structure similarity network computed from all-by-all pairwise comparisons of 170 nonredundant structures using the TM-Align algorithm. Edges represent pairwise structural alignments with a TMScore of at least 0.85 (main network) or at least 0.73 (inset). (A TM-Align score of 0.5 is considered statistically significant (72).) Nodes with similarity scores below these thresholds appear as disconnected from main clusters. Each circular node corresponds to a representative structure of the superfamily and is colored according to the SFLD subgroup to which it is assigned based on a careful curation protocol (34). Subgroups are muconate cycloisomerase (MLE), methylaspartate ammonia lyase (MAL), glucarate dehydratase (GlucD), mannonate dehydratase (ManD), enolase (Enol), galactarate dehydratase (GalD) and mandelate racemase (MR). Although all members of the superfamily share conserved active site machinery associated with the conserved fundamental partial reaction they all catalyze (28), they perform different overall chemical reactions using different substrates. Within the superfamily, while some subgroups are monofunctional and others include multiple reactions, each is named for a single experimentally characterized `founder’ reaction assigned to it by SFLD curators. For example, the MR subgroup includes the mandelate racemase reaction as well as many acid sugar dehydratases. The other subgroup containing several different overall reactions, the MLE subgroup, contains a reaction of the same name as well reactions that include dipeptide epimerases, n-acyl amino acid racemases and others. (B) Reaction similarity network computed from all-by-all pairwise comparisons of 33 overall superfamily reactions. Each square node represents a superfamily reaction colored according to the SFLD subgroup of the enzymes that catalyze it. Edges represent pairwise reaction center similarity scores of at least 0.17. The starred node designates a reaction found in both the MR and GalD subgroups, with three distinct families catalyzing the dehydration of galactarate (73–75). Labeled nodes designate reaction families discussed in the text or figures. The circle distinguishes enzymes from the MLE (syn) sub-subgroup, the members of which differ in substrate specificity rather than in overall reaction. (C) Substrate similarity network. Each hexagonal node corresponds to a known substrate involved in a reaction catalyzed by an enolase superfamily enzyme colored by the subgroup of the corresponding enzyme. Edges represent Tanimoto scores of at least 0.54. The starred node designates a substrate found in both the MR and GalD subgroups.

### Combining sequence, structure and chemical similarity to generate a more complete picture of enzyme structure–function relationships

Protein similarity networks (46) in which protein sequences or structures are used as nodes and pairwise similarities are used as edges were previously computed for core superfamilies at all levels of the SFLD hierarchy. The addition of reaction data in a chemical format to the SFLD provides the new information required for computing chemical similarity networks as well. As described in Methods, reaction or associated small molecule features were used as nodes in these networks and their pairwise similarities were used as edges. The formal addition of this chemical context allowed us to compare chemical similarity-based networks with networks depicting evolution-based similarity. For this proof-of-concept study, we compared the evolution- and chemistry-based functional classifications using the enolase superfamily for retrospective analysis. The next section describes the results of these comparisons. As the number of representative structures in the SFLD for the enolase superfamily (170 structures) tracks with the number of its known chemical reactions (33 reactions) at a more similar scale than do the number of sequences (48 847 unique sequences), structure similarity networks are provided in this paper to illustrate evolutionary relationships. Sequence similarity networks at all levels of the SFLD hierarchy are provided for download from the SFLD archive.

### Differences between evolutionary-based and the chemistry-based classification of enzymes complicate functional annotation

Comparison of the structure and chemical similarity networks for the enolase superfamily reveals profound differences between the evolutionary- and chemistry-based classification systems (Figure 4). The structure similarity network colored by the enolase superfamily subgroups defined from the evolutionary perspective is shown in Figure 4A. (The corresponding sequence similarity network is provided in Figure S4. It shows subgroup clustering relationships that are largely similar to those in the structure similarity network, although the sequence similarity network has much broader coverage of superfamily members than does the structure-based network.) The reaction similarity network calculated using the reaction center as the similarity metric (47) is shown in Figure 4B (see Methods for other chemical similarity metrics used for this work), while the substrate similarity network was calculated using a small molecule similarity metric is shown in Figure 4C. As with Figure 4A, Figures 4B and 4C are colored by subgroup designation to allow comparison of the evolutionary and chemical classification systems using the same annotation mappings. As the substrate similarity network provided in Figure 4C and the product similarity network (calculated but not shown) shows a subgroup clustering pattern that is similar to that of Figure 4B, only observations for Figure 4B are discussed further in this paper. The substrate and product similarity networks produced in this work are available for download from the SFLD Archive, however.

The structure similarity network (Figure 4A) confirms that structures from the same SFLD subgroup largely cluster together, as expected, since these subgroups are defined based on sequence, structural and active site similarity. Most of these subgroups are presumed to be monofunctional, i.e. enolase (Enol), and the acid sugar dehydratase subgroups glucarate dehydratase (GlucD), mannonate dehydratase (Man D) and galactarate dehydratase 2 (Gal D). The nodes representing the mandelate racemase (MR) and muconate cycloisomerase (MLE) subgroups also form cogent clusters in Figure 4A, although both of the latter are known to include multiple different overall reactions. Figure S5 shows the reactions and associated EC numbers of enzymes that span the main reaction types identified in the SFLD for the enolase superfamily. Figure S6 shows the structure similarity network mapped with annotations at the individual (monofunctional) family level. Here, individual reaction families of the MR subgroup are well resolved, providing a more informative view of their similarity relationships. For the enolase superfamily (and for others investigated in an earlier misannotation study (32)), annotation transfer at the family level is typically the most reliable starting point for transferring annotations from enzymes of known reaction specificity to unknowns. This is because sequences within reaction families tend to be more similar to each other than are sequences within their parent subgroups. Again, this is not surprising as families are defined in the ESFO as monofunctional groups of enzymes that catalyze the same reaction using a similar mechanism.

As with Figure 4A, the mapping of the family annotations for structures in the MLE subgroup (Figure S6) shows a tighter clustering than is represented for the MR subgroup, consistent with their greater intra-subgroup similarity. Interestingly, literature reports indicate catalytic promiscuity among some of the MLE subgroup enzymes (44, 48, 49). Additionally, an early engineering effort using single amino acid substitutions in a subset of MLE subgroup enzymes produced functional promiscuity among them (50).

Mapping of subgroup designations to the reaction similarity network is shown in Figure 4B. This figure shows that the multiple different reactions of the MR subgroup (light orange nodes) and the monofunctional acid sugar dehydratase subgroups largely cluster together in both Figures 4A and 4B. In contrast, the clustering of the nodes of the MLE subgroup (blue nodes) is clearly disparate, suggesting a substantial disconnect between the evolution and chemistry-based classifications for this subgroup. Similar types of disconnects were noted earlier in a small-scale study of enzymes from several different functionally diverse superfamilies (12) as well as in a larger analysis of misannotation errors in the enolase and several other superfamilies (32). This latter study found that in the enolase superfamily, reaction families of the MR and MLE subgroups were routinely misannotated in major databases, either due to annotation of unknowns to an incorrect enolase superfamily reaction or as a `mandelate racemase/muconate lactonizing enzyme’. This latter annotation is especially misleading as neither the literature nor our rather deep understanding of the enolase superfamily suggests that an enzyme exists in nature that catalyzes both of these reactions.

One reason for the occurrence of the high levels of misannotation in functionally diverse superfamilies described in the references listed above is that while the known superfamily reactions such as those from the enolase superfamily all `look alike’ from a sequence and structural perspective, their known overall reactions can differ widely, as shown by their highly varied EC numbers (Figure S5). Moreover, both the order in which the functions of these enzymes have been experimentally annotated in public databases and the extent of their sequence and structural similarities make it challenging to determine accurate cut-offs for annotation transfer of EC numbers to uncharacterized proteins in the absence of biochemical confirmation.

Although estimates of general similarity thresholds have been proposed at which annotation transfer is likely to be correct (for example, (51, 52)), the results from our analyses of the networks suggest that except for highly similar sequences in functionally diverse enzyme superfamilies this assumption is problematic. It appears instead that each superfamily may be substantially unique with respect to how its varied chemical capabilities have emerged through divergent changes from an ancestral structural scaffold (53). Thus, general similarity thresholds may be inadequate for accurate annotation transfer for the divergent proteins that typify functionally diverse enzyme superfamilies (see, for example, (12, 32, 48, 54)).

Even at the family level of annotation (Figure S6), our study shows differences between the evolutionary-based classification (Figure 4A) and the chemistry-based view (Figures 4B and 4C). Function assignment for the *o*-succinylbenzoate synthase (OSBS) enzyme family provides an informative example of this disconnect. From the sequence and structural perspective, the OSBS family enzymes can be confidently assigned to the MLE subgroup based on several lines of evidence (for example, (28,48)). From the chemical perspective (Figure 4B), however, the OSBS family enzymes cluster more closely in the network with the MR subgroup. This observation has been rationalized in part due to the more `general’ features of the active sites of OSBS-catalyzing enzymes relative to other reaction families in the enolase superfamily (48). The `generalist’ features of proteins have been noted by others as well (for examples, see (55, 56)). Other complex features of OSBS structure and mechanism also contribute to the disconnect between its evolutionary- and chemistry-based classifications*.*

### Mapping of structure and chemical similarity networks by EC number

As with the mappings by evolution-based subgroup annotations onto the structure and chemical similarity networks, mapping the enzymes represented by the first three digits of their EC numbers illustrates additional disconnects between the evolution-based and chemistry-based classifications. For example, the green nodes in Figures S7A and S7B represent the same overall reaction (EC 4.2.1), which designates them as catalyzing hydro-lyase chemistry. While these nodes cluster well together in the reaction similarity network shown in Figure S7B, they are disparate across the structure similarity network shown in Figure S7A and are assigned to several different and highly divergent subgroups (Figure 4). A better understanding of this superfamily-wide disconnect is difficult to interpret at this time as the paths by which the enzymes of each subgroup evolved are poorly understood. Although a high confidence phylogenetic tree would be helpful in achieving this goal, the extreme divergence of these subgroups currently prevents its calculation of a high confidence phylogenetic tree that could aid our understanding these observations.

The OSBS reaction offers a specific example of this disconnect. While OSBS is appropriately assigned to EC 4.2.1 based on the chemical transformation it catalyzes (Figure S7B), its evolution-based metrics and previous literature (48) clearly assign OSBS to the MLE subgroup (Figure 4). Moreover, nearly all the known reactions of enzymes in the MLE subgroup are assigned to an EC class and subclass (EC 5.1 or 5.5) that differs from the EC 4.2 subclass of OSBS (see Table S1). Other more recent work has provided new insight for understanding this intriguing result (57–59).

As with the differences between the subgroup and family level mappings for the structure-based networks (Figures 4 and S6), the networks mapped with EC classifications track better with the evolution-based classifications at the family level than they do at the less granular subgroup level. This trend is reflected in Figure S8, which details how individual reaction families map to the structure and reaction similarity networks for each of the three main EC number groupings represented in the enolase superfamily. For example, the majority of green nodes associated with EC 4.2.1 in the structure similarity network (Figure S8A**)** represents acid sugar dehydratases of the Enol, GalD, GlucD, ManD and the MR subgroups. As with Figure S7, the OSBS reaction family remains an outlier in Figure S8, consistent with the results discussed above for Figure S7.

Highlighting yet another inconsistency between the evolutionary and chemical perspectives, the mandelate racemase reaction family (EC 5.1.2, magenta node in Figure S8D), which belongs to the MR subgroup from an evolutionary perspective (28) is connected in the chemical similarity network shown in Figure S7B to the purple node labeled as an N-succinyl amino acid racemase (NSAR) (EC 5.1.1), which belongs to the MLE subgroup. Thus, from the chemical perspective, the mandelate racemase reaction clusters with reactions of the NSARs and dipeptide epimerases of the MLE subgroup (Figure S8D), rather than with the acid sugar dehydratases. This pattern is in disagreement with evidence from sequence and structure similarity that assigns the mandelate racemase family to the MR subgroup for which it is the namesake reaction. A substantial literature supports assignment of mandelate racemase to the MR subgroup based on overall and active site similarity (see for overviews of this literature (28, 60)).

### Summary and future directions

For the enolase superfamily, the results of this study illustrate several of the conceptually distinct ways in which structure- and chemistry-based classifications of enzyme function, as well as EC number designations investigated in this work show profound disconnects. The enolase superfamily networks and those calculated in the SFLD for the haloacid dehalogenase and radical SAM superfamilies show related types of disconnects. Specific examples of similar trends have been published previously regarding members of the radical SAM superfamily (30, 61); the Radical SAM similarity networks available from the SFLD Archive offer an additional data resource for interpretation of those reports.

This report illustrates in a new way fundamental and complex differences between evolution- and chemistry-based classifications of enzyme function although each is appropriate for use in the respective context for which each they were developed. Using a retrospective analysis of a well-studied enzyme superfamily, we show that our approach for accurate annotation transfer from known reactions to unknown homologs could be enlightened by examining these two classifications together and on the scale of large superfamilies. These results also support our previous observations regarding some of the hazards in attempting to define general similarity thresholds appropriate for annotation transfer from chemical classifications to homologous unknowns. Automated investigation of many more superfamilies will be needed to evaluate the general utility of our findings for the broader universe of functionally diverse enzyme superfamilies.

The proof-of-concept strategy described in this work was designed as a first step in enabling new types of comparisons between enzyme classifications based on evolutionary and chemical perspectives. In providing the concept representations and controlled vocabularies required to link these data in a computationally useful manner, it aims to inform new work by others to extend the scale and depth at which these types of comparisons can be mounted. Key to the MEERCat strategy, linking together the previously developed evolutionary-based structure–function ESFO ontology with the chemistry-based EMO ontology provides an example of one way to enable computing with both types of classification together.

While this report focuses on the application of our strategy to functionally diverse enzyme superfamilies, similar approaches could be developed for general application to broader use cases that also could benefit from using the reaction- and the protein-centric points of view together. For example, future extensions to this approach could be developed for investigation of convergent evolution of particular functions in different superfamilies and fold classes.

## Materials and Methods

### Representation of chemical entities

ChEBI was chosen for describing the chemistry of the overall transformation because it contains molecular data in several different formats and can be freely downloaded and stored locally.

Besides inclusion of the overall reaction as a key chemical descriptor of the chemistry an enzyme performs, the MEERCat strategy captures more complex information about reaction mechanism using the reaction scheme concept (not shown here; see (41)). Minimally, this concept includes at least the overall reaction and its links to specific EFDs. Alternatively, it can also represent more detailed information, giving a richer description of the chemistry that occurs. This may include such features as catalytic residues, directionality and cofactors. The full schema for reaction representation implemented in the SFLD using the MEERCat strategy is shown in Figure S2.

Reaction mapping, similarity calculations and annotation are done using the Reaction Decoder Tool (RDT) (47). Small molecule mapping and similarity calculations were done using the Small Molecule Subgraph Detector (SMSD) toolkit (62). Both are designed to use MDL file formats (.mol and .rxn) as well as SMILES strings (63). Both SMILES and .mol formats are stored in ChEBI. The reactions are built from the .mol files in the .rxn format defined in the MDL file specifications. Reactions are visualized by taking the constituent molecules and using RDKit (64) to convert the SMILES strings into 2D representations that are then shown on the Archive website.

The program used to compare two reactions, ReactionDecoder (ReactionDecoder.jar), was executed using the default parameters to compare .rxn files. The results were written to a file in the same directory as the query and target files. A Linux 2.6 host was used with a local compute cluster using Java version is 1.8 (also known as Java 8).

### Similarity networks

Similarity networks generated for the enolase superfamily were computed using algorithms inspired by Pythoscape (65), tailored to work with the available computing infrastructure. Visualization of networks are laid out as thresholded networks (46) using the yFiles Organic layout algorithm as implemented in Cytoscape 3 (66). Lengths of edges are not meaningful except that sequences in tightly clustered groups are relatively more similar to each other than sequences with few connections. Thresholded networks were used to explore qualitative differences between protein structural- and chemical-similarities for biochemically and structurally characterized enzymes. This was done by mapping SFLD functional annotations (as well as EC number designations for overall reactions) for subgroups and families of the enolase superfamily onto structure and chemical similarity networks. While the similarities among each type of network represent quantitative scores as determined by each algorithm used to compute similarities, the thresholds for drawing edges (lines) between nodes in the structural similarity networks were chosen by qualitative inspection to effectively enable visualization. For structure similarity networks, thresholds were chosen to enable visualization of connectivity among the most similar subgroups, leaving the most dissimilar subgroups disconnected. The threshold for drawing edges between nodes in the chemical similarity networks were chosen similarly, with the aim of visualizing the best connected reactions comprising each subgroup annotated by reaction similarity or small molecule similarity. For all the network figures presented in the main text and supplementary figures, the algorithms used, the chosen thresholds and other details are described in each figure legend.

### Sequence similarity network

The sequence similarity network for the enolase superfamily was computed and laid out similarly to those computed for structural similarity networks.

### Structure similarity network

A set of representative enolase superfamily structures was gathered so as to include a single structure for each unique sequence for which a structure was available in the PDB (67) as of 3 November 2017. Where multiple structures were available for a given sequence, a single representative was chosen with a preference for wild type, liganded structures missing the fewest number of residues. A structure similarity network was computed from all-by-all pairwise comparisons of these 170 nonredundant structures using the TM-Align algorithm (68) using scripts similar to those used for generation of sequence similarity networks in the SFLD. TM-align scores of 0.73 and 0.85 were chosen as the threshold cutoffs for drawing edges and produced 9460 and 2697 edges, respectively.

### Chemical similarity network

These include overall reaction and molecular similarity networks (substrates, products and all molecules in a reaction) for the superfamilies and subgroups for which more than three reactions are annotated.

Reaction similarity network. Networks were generated comprising the 33 reactions from the enolase superfamily for which the SFLD contained reaction data in .rxn format. For each pair of reactions, a comparison was made using the RDT. The scoring threshold for drawing edges was chosen empirically to allow useful visualization of reaction similarity patterns. .rxn files were staged on the SFLD host and the reaction comparisons were calculated by a multi-node compute cluster with access to the .rxn files. Resulting edges were stored in a MySQL database keyed by the hash of the reaction IDs of the pair of reactions that share the edge.

Reaction similarity was calculated (and stored) three ways: reaction center, bond change and small molecule similarity using the EC-BLAST tool (69). The reaction center similarity metric provides the most nuanced view of overall reaction similarity as it includes information on both bond changes and the atomic environment of the bonds and atoms at which the changes occur.

Reaction center similarity. The similarity between two reactions can be calculated based on fingerprints. The size of the computed fingerprints is dynamic as it depends on the number of reaction patterns in each reaction as well as the method of similarity used. The reaction center metric is based on a comparison of the reactive centers of the reactions. First, atom–atom mapping is calculated. For this step, changes involved in the transformation are encoded using a circular fingerprint, which enables the description of the chemical environment around the atoms of interest. Figure S9 provides an example illustrating a reaction center and accompanying bond changes for muconate cycloisomerase (EC 5.5.1.1).

Bond change similarity is based on a comparison of the bond changes (bonds formed/cleaved, order changes and stereo changes). Following atom–atom mapping, bond changes are encoded as a fingerprint that is used to calculate similarity. Bond change similarities for this work are not shown.

Small molecule similarity is based on comparison of the chemical structure of the small molecule moieties in the reactions. The chemical similarity of the full structure of small molecules used as substrate(s) and product(s) are calculated using the fingerprint generation methods in Small Molecule Subgraph Detector (SMSD) toolkit (62) from which a standard Tanimoto similarity (70) is calculated.

### Small molecule similarity network

These were computed using an analogous method to that for the reaction similarity network, except that the SMSD toolkit was used as the comparison tool. Data gathered for computing the small molecule similarity network included the associated SMILES strings to enable the use of Cytoscape’s ChemViz2 Plugin (71) for analysis of the networks.

## Supplementary Material

MEERCat_SuppInfo_format-11_2_19

## References

[1] Ashburner M., Ball C.A., Blake J.A. et al. (2000) Gene ontology: tool for the unification of biology. The Gene Ontology Consortium. Nat. Genet., 25, 25–29. 10802651 10.1038/75556 PMC3037419

[2] Kramarz B. and Lovering R.C. (2019) Gene ontology: a resource for analysis and interpretation of Alzheimer's disease data In: Wisniewski T (ed). Alzheimer's Disease, Codon Publications, Brisbane. 31895510

[3] Zhou N., Jiang Y., Bergquist T.R. et al. (2019) The CAFA challenge reports improved protein function prediction and new functional annotations for hundreds of genes through experimental screens. Genome Biol., 20, 244. 31744546 10.1186/s13059-019-1835-8 PMC6864930

[4] Tipton K.F. (1992) Enzyme Nomenclature: Recommendations of the Nomenclature Committee of the International Union of Biochemistry and Molecular Biology (IUBMB). NC-IUBMB, New York. 10.1111/j.1432-1033.1994.tb18960.x 7957164

[5] McDonald A.G. and Tipton K.F. (2014) Fifty-five years of enzyme classification: advances and difficulties. FEBS J., 281, 583–592. 24103004 10.1111/febs.12530

[6] Jeske L., Placzek S., Schomburg I. et al. (2019) BRENDA in 2019: a European ELIXIR core data resource. Nucleic Acids Res., 47, D542–D549. 30395242 10.1093/nar/gky1048 PMC6323942

[7] Wittig U., Rey M., Weidemann A. et al. (2018) SABIO-RK: an updated resource for manually curated biochemical reaction kinetics. Nucleic Acids Res., 46, D656–D660. 29092055 10.1093/nar/gkx1065 PMC5753344

[8] Kanehisa M. (2017) Enzyme annotation and metabolic reconstruction using KEGG. Methods Mol. Biol., 1611, 135–145. 28451977 10.1007/978-1-4939-7015-5_11

[9] Kanehisa M., Furumichi M., Tanabe M. et al. (2017) KEGG: new perspectives on genomes, pathways, diseases and drugs. Nucleic Acids Res., 45, D353–D361. 27899662 10.1093/nar/gkw1092 PMC5210567

[10] Caspi R., Billington R., Keseler I.M. et al. (2020) The MetaCyc database of metabolic pathways and enzymes - a 2019 update. Nucleic Acids Res., 48, D445–D453. 31586394 10.1093/nar/gkz862 PMC6943030

[11] UniProt Consortium (2019) UniProt: a worldwide hub of protein knowledge. Nucleic Acids Res., 47, D506–D515. 30395287 10.1093/nar/gky1049 PMC6323992

[12] Babbitt P.C. (2003) Definitions of enzyme function for the structural genomics era. Curr. Opin. Chem. Biol., 7, 230–237. 12714057 10.1016/s1367-5931(03)00028-0

[13] Rigden D.J. and Fernandez X.M. (2019) The 26th annual Nucleic Acids Research database issue and Molecular Biology Database Collection. Nucleic Acids Res., 47, D1–D7. 30626175 10.1093/nar/gky1267 PMC6323895

[14] Furnham N., Dawson N.L., Rahman S.A. et al. (2016) Large-scale analysis exploring evolution of catalytic machineries and mechanisms in enzyme superfamilies. J. Mol. Biol., 428,253–267. 26585402 10.1016/j.jmb.2015.11.010 PMC4751976

[15] Martinez Cuesta S., Rahman S.A., Furnham N. et al. (2015) The classification and evolution of enzyme function. Biophys. J., 109, 1082–1086. 25986631 10.1016/j.bpj.2015.04.020 PMC4576142

[16] Tyzack J.D., Furnham N., Sillitoe I. et al. (2017) Understanding enzyme function evolution from a computational perspective. Curr. Opin. Struct. Biol., 47, 131–139. 28892668 10.1016/j.sbi.2017.08.003

[17] Sillitoe I., Dawson N., Lewis T.E. et al. (2019) CATH: expanding the horizons of structure-based functional annotations for genome sequences. Nucleic Acids Res., 47, D280–D284. 30398663 10.1093/nar/gky1097 PMC6323983

[18] Furnham N., Sillitoe I., Holliday G.L. et al. (2012) FunTree: a resource for exploring the functional evolution of structurally defined enzyme superfamilies. Nucleic Acids Res., 40, D776–D782. 22006843 10.1093/nar/gkr852 PMC3245072

[19] Tyzack J.D., Furnham N., Sillitoe I. et al. (2019) Exploring enzyme evolution from changes in sequence, structure, and function. Methods Mol. Biol., 1851, 263–275. 30298402 10.1007/978-1-4939-8736-8_14

[20] Akiva E., Brown S., Almonacid D.E. et al. (2014) The Structure-Function Linkage Database. Nucleic Acids Res., 42, D521–D530. 24271399 10.1093/nar/gkt1130 PMC3965090

[21] Ribeiro A.J.M., Holliday G.L., Furnham N. et al. (2018) Mechanism and Catalytic Site Atlas (M-CSA): a database of enzyme reaction mechanisms and active sites. Nucleic Acids Res., 46, D618–D623. 29106569 10.1093/nar/gkx1012 PMC5753290

[22] Swainston N., Batista-Navarro R., Carbonell P. et al. (2017) biochem4j: integrated and extensible biochemical knowledge through graph databases. PLoS One, 12, e0179130. 28708831 10.1371/journal.pone.0179130 PMC5510799

[23] Holliday G.L., Andreini C., Fischer J.D. et al. (2012) MACiE: exploring the diversity of biochemical reactions. Nucleic Acids Res., 40, D783–D789. 22058127 10.1093/nar/gkr799 PMC3244993

[24] Furnham N., Holliday G.L., Beer T.A. de et al. (2013) The Catalytic Site Atlas 2.0: cataloging catalytic sites and residues identified in enzymes. Nucleic Acids Res., 42, D485–D489. 24319146 10.1093/nar/gkt1243 PMC3964973

[25] Lombardot T., Morgat A., Axelsen K.B. et al. (2019) Updates in Rhea: SPARQLing biochemical reaction data. Nucleic Acids Res., 47, D596–D600. 30272209 10.1093/nar/gky876 PMC6324061

[26] Hastings J., Owen G., Dekker A. et al. (2016) ChEBI in 2016: improved services and an expanding collection of metabolites. Nucleic Acids Res., 44, D1214–D1219. 26467479 10.1093/nar/gkv1031 PMC4702775

[27] Madeira F., Park Y.M., Lee J. et al. (2019) The EMBL-EBI search and sequence analysis tools APIs in 2019. Nucleic Acids Res., 47, W636–W641. 30976793 10.1093/nar/gkz268 PMC6602479

[28] Babbitt P.C., Hasson M.S., Wedekind J.E. et al. (1996) The enolase superfamily: a general strategy for enzyme-catalyzed abstraction of the alpha-protons of carboxylic acids. Biochemistry, 35, 16489–16501. 8987982 10.1021/bi9616413

[29] Gerlt J.A. and Babbitt P.C. (2001) Divergent evolution of enzymatic function: mechanistically diverse superfamilies and functionally distinct suprafamilies. Annu. Rev. Biochem., 70, 209–246. 11395407 10.1146/annurev.biochem.70.1.209

[30] Holliday G.L., Akiva E., Meng E.C. et al. (2018) Atlas of the radical SAM superfamily: divergent evolution of function using a "plug and play" domain. Methods Enzymol., 606, 1–71. 30097089 10.1016/bs.mie.2018.06.004 PMC6445391

[31] Nagano N., Orengo C.A. and Thornton J.M. (2002) One fold with many functions: the evolutionary relationships between TIM barrel families based on their sequences, structures and functions. J. Mol. Biol., 321, 741–765. 12206759 10.1016/s0022-2836(02)00649-6

[32] Schnoes A.M., Brown S.D., Dodevski I. et al. (2009) Annotation error in public databases: misannotation of molecular function in enzyme superfamilies. PLoS Comput. Biol., 5, e1000605. 20011109 10.1371/journal.pcbi.1000605 PMC2781113

[33] Mitchell A.L., Attwood T.K., Babbitt P.C. et al. (2019) InterPro in 2019: improving coverage, classification and access to protein sequence annotations. Nucleic Acids Res., 47, D351–D360. 30398656 10.1093/nar/gky1100 PMC6323941

[34] Holliday G.L., Brown S.D., Akiva E. et al. (2017) Biocuration in the structure-function linkage database: the anatomy of a superfamily. Database (Oxford), 2017, 1–12. 10.1093/database/bax006 PMC5467563 28365730

[35] Almonacid D.E. and Babbitt P.C. (2011) Toward mechanistic classification of enzyme functions. Curr. Opin. Chem. Biol., 15, 435–442. 21489855 10.1016/j.cbpa.2011.03.008 PMC3551611

[36] Chiang R.A., Sali A. and Babbitt P.C. (2008) Evolutionarily conserved substrate substructures for automated annotation of enzyme superfamilies. PLoS Comput. Biol., 4, e1000142. 18670595 10.1371/journal.pcbi.1000142 PMC2453236

[37] El-Gebali S., Mistry J., Bateman A. et al. (2019) The Pfam protein families database in 2019. Nucleic Acids Res., 47, D427–D432. 30357350 10.1093/nar/gky995 PMC6324024

[38] Brown S.D., Gerlt J.A., Seffernick J.L. et al. (2006) A gold standard set of mechanistically diverse enzyme superfamilies. Genome Biol., 7, R8. 16507141 10.1186/gb-2006-7-1-r8 PMC1431709

[39] Weininger D. (1988) SMILES, a chemical language and information system. 1. Introduction to methodology and encoding rules. J. Chem. Inf. Model., 28, 31–36.

[40] Pegg S.C., Brown S.D., Ojha S. et al. (2006) Leveraging enzyme structure-function relationships for functional inference and experimental design: the structure-function linkage database. Biochemistry, 45, 2545–2555. 16489747 10.1021/bi052101l

[41] Holliday G.L., Murray-Rust P. and Rzepa H.S. (2006) Chemical markup, XML, and the world wide web. 6. CMLReact, an XML vocabulary for chemical reactions. J. Chem. Inf. Model., 46, 145–157. 16426051 10.1021/ci0502698

[42] Babbitt P.C. and Gerlt J.A. (1997) Understanding enzyme superfamilies. Chemistry as the fundamental determinant in the evolution of new catalytic activities. J. Biol. Chem., 272, 30591–30594. 9388188 10.1074/jbc.272.49.30591

[43] Fleischmann A., Darsow M., Degtyarenko K. et al. (2004) IntEnz, the integrated relational enzyme database. Nucleic Acids Res., 32, D434–D437. 14681451 10.1093/nar/gkh119 PMC308853

[44] Sakai A., Fedorov A.A., Fedorov E.V. et al. (2009) Evolution of enzymatic activities in the enolase superfamily: stereochemically distinct mechanisms in two families of cis,cis-muconate lactonizing enzymes. Biochemistry, 48, 1445–1453. 19220063 10.1021/bi802277h PMC2746992

[45] Burroughs A.M., Allen K.N., Dunaway-Mariano D. et al. (2006) Evolutionary genomics of the HAD superfamily: understanding the structural adaptations and catalytic diversity in a superfamily of phosphoesterases and allied enzymes. J. Mol. Biol., 361, 1003–1034. 16889794 10.1016/j.jmb.2006.06.049

[46] Atkinson H.J., Morris J.H., Ferrin T.E. et al. (2009) Using sequence similarity networks for visualization of relationships across diverse protein superfamilies. PLoS One, 4, e4345. 19190775 10.1371/journal.pone.0004345 PMC2631154

[47] Rahman S.A., Torrance G., Baldacci L. et al. (2016) Reaction Decoder Tool (RDT): extracting features from chemical reactions. Bioinformatics, 32, 2065–2066. 27153692 10.1093/bioinformatics/btw096 PMC4920114

[48] Glasner M.E., Fayazmanesh N., Chiang R.A. et al. (2006) Evolution of structure and function in the o-succinylbenzoate synthase/N-acylamino acid racemase family of the enolase superfamily. J. Mol. Biol., 360, 228–250. 16740275 10.1016/j.jmb.2006.04.055

[49] Song L., Kalyanaraman C., Fedorov A.A. et al. (2007) Prediction and assignment of function for a divergent N-succinyl amino acid racemase. Nat. Chem. Biol., 3, 486–491. 17603539 10.1038/nchembio.2007.11

[50] Schmidt D.M., Mundorff E.C., Dojka M. et al. (2003) Evolutionary potential of (beta/alpha)8-barrels: functional promiscuity produced by single substitutions in the enolase superfamily. Biochemistry, 42, 8387–8393. 12859183 10.1021/bi034769a

[51] Tian W. and Skolnick J. (2003) How well is enzyme function conserved as a function of pairwise sequence identity? J. Mol. Biol., 333, 863–882. 14568541 10.1016/j.jmb.2003.08.057

[52] Todd A.E., Orengo C.A. and Thornton J.M. (2001) Evolution of function in protein superfamilies, from a structural perspective. J. Mol. Biol., 307, 1113–1143. 11286560 10.1006/jmbi.2001.4513

[53] Brown S.D. and Babbitt P.C. (2014) New insights about enzyme evolution from large scale studies of sequence and structure relationships. J. Biol. Chem., 289, 30221–30228. 25210038 10.1074/jbc.R114.569350 PMC4215206

[54] Seffernick J.L., Souza M.L. de, Sadowsky M.J. et al. (2001) Melamine deaminase and atrazine chlorohydrolase: 98 percent identical but functionally different. J. Bacteriol., 183, 2405–2410. 11274097 10.1128/JB.183.8.2405-2410.2001 PMC95154

[55] Khersonsky O. and Tawfik D.S. (2010) Enzyme promiscuity: a mechanistic and evolutionary perspective. Annu. Rev. Biochem., 79, 471–505. 20235827 10.1146/annurev-biochem-030409-143718

[56] Glasner M.E., Gerlt J.A. and Babbitt P.C. (2007) Mechanisms of protein evolution and their application to protein engineering. Adv. Enzymol. Relat. Areas Mol. Biol., 75, 193–239 xii–xiii. 17124868 10.1002/9780471224464.ch3

[57] Brizendine A.M., Odokonyero D., McMillan A.W. et al. (2014) Promiscuity of Exiguobacterium sp. AT1b o-succinylbenzoate synthase illustrates evolutionary transitions in the OSBS family. Biochem. Biophys. Res. Commun., 450, 679–684. 24937446 10.1016/j.bbrc.2014.06.034

[58] Odokonyero D., Ragumani S., Lopez M.S. et al. (2013) Divergent evolution of ligand binding in the o-succinylbenzoate synthase family. Biochemistry, 52, 7512–7521. 24060347 10.1021/bi401176d PMC3908897

[59] Zhu W.W., Wang C., Jipp J. et al. (2012) Residues required for activity in Escherichia coli o-succinylbenzoate synthase (OSBS) are not conserved in all OSBS enzymes. Biochemistry, 51, 6171–6181. 22775324 10.1021/bi300753j

[60] Gerlt J.A., Babbitt P.C. and Rayment I. (2005) Divergent evolution in the enolase superfamily: the interplay of mechanism and specificity. Arch. Biochem. Biophys., 433, 59–70. 15581566 10.1016/j.abb.2004.07.034

[61] Betz J.N., Boswell N.W., Fugate C.J. et al. (2015) [FeFe]-hydrogenase maturation: insights into the role HydE plays in dithiomethylamine biosynthesis. Biochemistry, 54, 1807–1818. 25654171 10.1021/bi501205e PMC4839199

[62] Rahman S.A., Bashton M., Holliday G.L. et al. (2009) Small Molecule Subgraph Detector (SMSD) toolkit. J. Chem., 1, 12. 10.1186/1758-2946-1-12 PMC2820491 20298518

[63] Weininger D., Weininger A. and Weininger J.L. (1989) SMILES.2. Algorithm for generation of unique SMILES notation. J. Chem. Inf. Comput. Sci., 29, 97–101.

[64] Landrum G. (2006) RDKit: open-source cheminformatics. Online, 3, 2012.

[65] Barber A.E.I.I. and Babbitt P.C. (2012) Pythoscape: a framework for generation of large protein similarity networks. Bioinformatics. 28, 2845–2846. 22962345 10.1093/bioinformatics/bts532 PMC3476340

[66] Shannon P., Markiel A., Ozier O. et al. (2003) Cytoscape: a software environment for integrated models of biomolecular interaction networks. Genome Res., 13, 2498–2504. 14597658 10.1101/gr.1239303 PMC403769

[67] Burley S.K., Berman H.M., Bhikadiya C. et al. (2019) RCSB Protein Data Bank: biological macromolecular structures enabling research and education in fundamental biology, biomedicine, biotechnology and energy. Nucleic Acids Res., 47, D464–D474. 30357411 10.1093/nar/gky1004 PMC6324064

[68] Zhang Y. and Skolnick J. (2005) TM-align: a protein structure alignment algorithm based on the TM-score. Nucleic Acids Res., 33, 2302–2309. 15849316 10.1093/nar/gki524 PMC1084323

[69] Rahman S.A., Cuesta S.M., Furnham N. et al. (2014) EC-BLAST: a tool to automatically search and compare enzyme reactions. Nat. Methods, 11, 171–174. 24412978 10.1038/nmeth.2803 PMC4122987

[70] Rogers D.J. and Tanimoto T.T. (1960) A computer program for classifying plants. Science, 132, 1115–1118. 17790723 10.1126/science.132.3434.1115

[71] Saito R., Smoot M.E., Ono K. et al. (2012) A travel guide to Cytoscape plugins. Nat. Methods, 9, 1069–1076. 23132118 10.1038/nmeth.2212 PMC3649846

[72] Xu J. and Zhang Y. (2010) How significant is a protein structure similarity with TM-score = 0.5? Bioinformatics, 26, 889–895. 20164152 10.1093/bioinformatics/btq066 PMC2913670

[73] Groninger-Poe F.P., Bouvier J.T., Vetting M.W. et al. (2014) Evolution of enzymatic activities in the enolase superfamily: galactarate dehydratase III from Agrobacterium tumefaciens C58. Biochemistry, 53, 4192–4203. 24926996 10.1021/bi5005377 PMC4081050

[74] Rakus J.F., Kalyanaraman C., Fedorov A.A. et al. (2009) Computation-facilitated assignment of the function in the enolase superfamily: a regiochemically distinct galactarate dehydratase from Oceanobacillus iheyensis. Biochemistry, 48, 11546–11558. 19883118 10.1021/bi901731c PMC2787699

[75] Yew W.S., Fedorov A.A., Fedorov E.V. et al. (2007) Evolution of enzymatic activities in the enolase superfamily: L-talarate/galactarate dehydratase from salmonella typhimurium LT2. Biochemistry, 46, 9564–9577. 17649980 10.1021/bi7008882

