# Supplementary material for: A strategy for large-scale comparison of evolutionary- and reaction-based classifications of enzyme function
Source: Database (Oxford). 2020 May 25;2020:baaa034. doi: 10.1093/database/baaa034 (PMC7246345; doi:10.1093/database/baaa034)
Supplement: MEERCat_SuppInfo_format-11_2_19 [file meercat_suppinfo_format-11_2_19.pdf]

Supporting Information

A strategy for large-scale comparison of evolutionary- and reaction-based classifications of enzyme function

Gemma L. Holliday[1,4*], Shoshana Brown[1], David Mischel[1], Benjamin J. Polacco[1], and Patricia Babbitt [1,2,3*]

[1]Department of Bioengineering and Therapeutic Sciences, University of California, San Francisco, San Francisco, CA 94143, USA.

[2]Department of Pharmaceutical Chemistry, University of California, San Francisco, San Francisco, CA 94143, USA.

[3]Quantitative Biosciences Institute, University of California, San Francisco, San Francisco, CA 94158, USA.

[4]Current Address: Medicines Discovery Catapult, Mereside, Alderley Park, Alderley Edge SK10 4TG, United Kingdom

[*] Corresponding authors

Correspondence:
Gemma L. Holliday, gemma.l.holliday@gmail.com
Patricia C. Babbitt, babbitt@cgl.ucsf.edu.

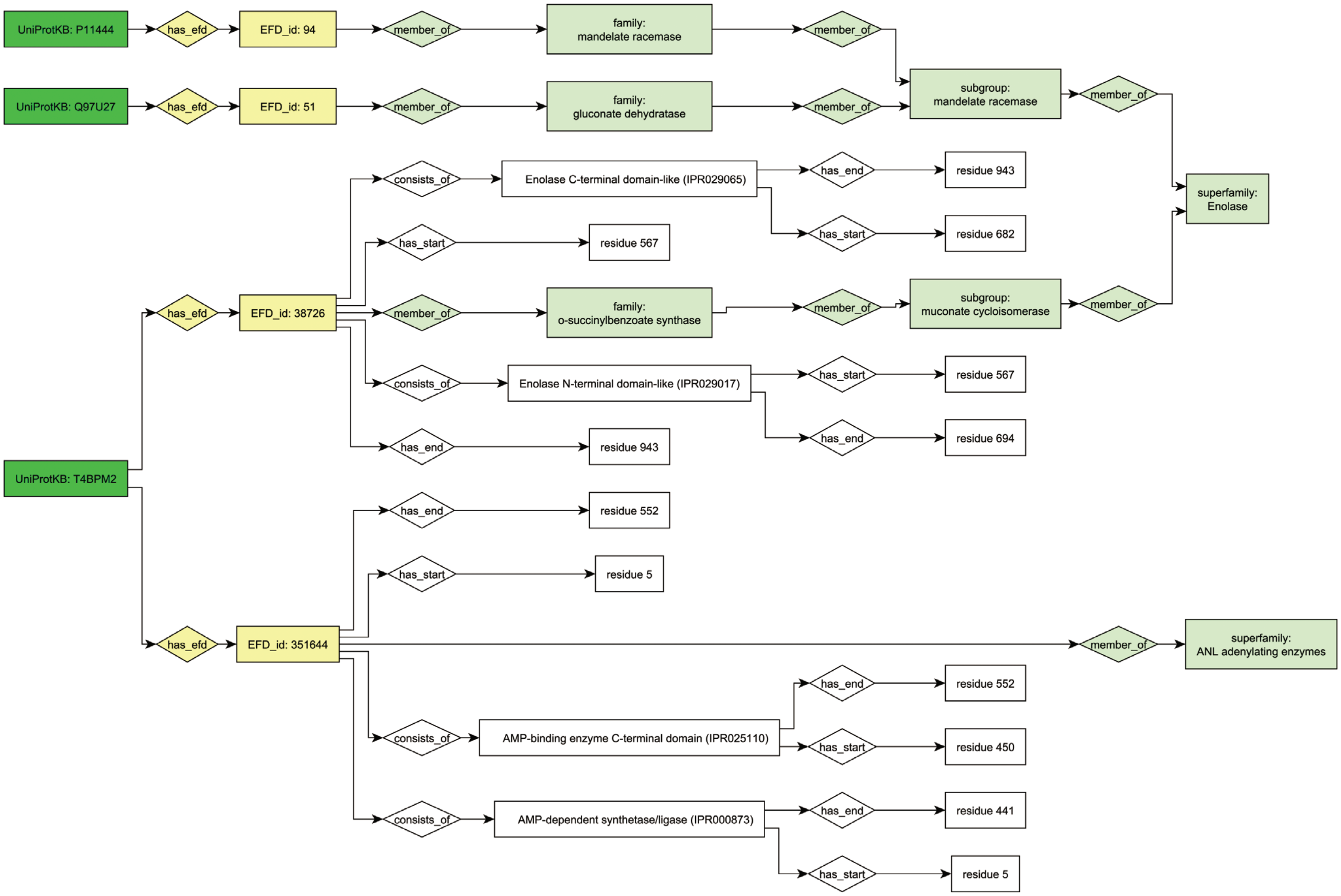

Figure S1. Relationships between proteins and EFDs in the SFLD hierarchy. Reading from left to right, the figure illustrates how a specific protein (darker green rectangle) relates to the EFD (pale yellow) and how those EFDs map to the SFLD hierarchy (pale green). Top: Proteins named by their UniProtKB accession numbers, P11444 and Q97U27, represent EFDs for two different reaction families (mandelate racemase (EFD 94) and gluconate dehydratase (EFD 51), respectively) in the Enolase Superfamily, both of which belong to the Mandelate racemase subgroup. (In the SFLD, mandelate racemase is the name of both a family and a subgroup.) For simplicity, their domain structures and characteristics (designated in Pfam by separate N-terminal and C-terminal domain models) are not shown. For the more complex UniProtKB protein, T4BPM2, details of its domain structure are shown as this enzyme is comprised of two nonhomologous EFDs, EFD_id:38726 and EFD_id: 351644, each of which is a member of a different superfamily as described by the InterPro (1) identifiers for the Enolase superfamily (C-terminal domain InterPro (IPR) accession: 029065/N-terminal domain IPR:029017) and the ANL adenylating enzymes superfamily (includes acyl- and aryl-CoA synthetases, firefly luciferase, and the adenylation domains of the modular non-ribosomal peptide synthetases (2), AMP-binding enzyme C-terminal domain IPR025110/ AMP-dependent synthetase/ligase IPR:000873), respectively. (The InterPro domain information is not explicitly included in the SFLD ESFO and is only shown in this example for clarity.) The white terms and relationships show the details available for these EFDs, including the start and stop positions and the different domains that comprise them. For UniProtKB protein T4BPM2, the EFD from the Enolase Superfamily (EFD_id:38726) is assigned to the o-succinylbenzoate synthase family. Its two domains required for function, the N- and C-terminal domains, are shown. The second EFD of T4BPM2, EFD_id:351644, is listed only as a member of the ANL adenylating enzymes superfamily as there are no curated subgroups or families for this superfamily in the SFLD.

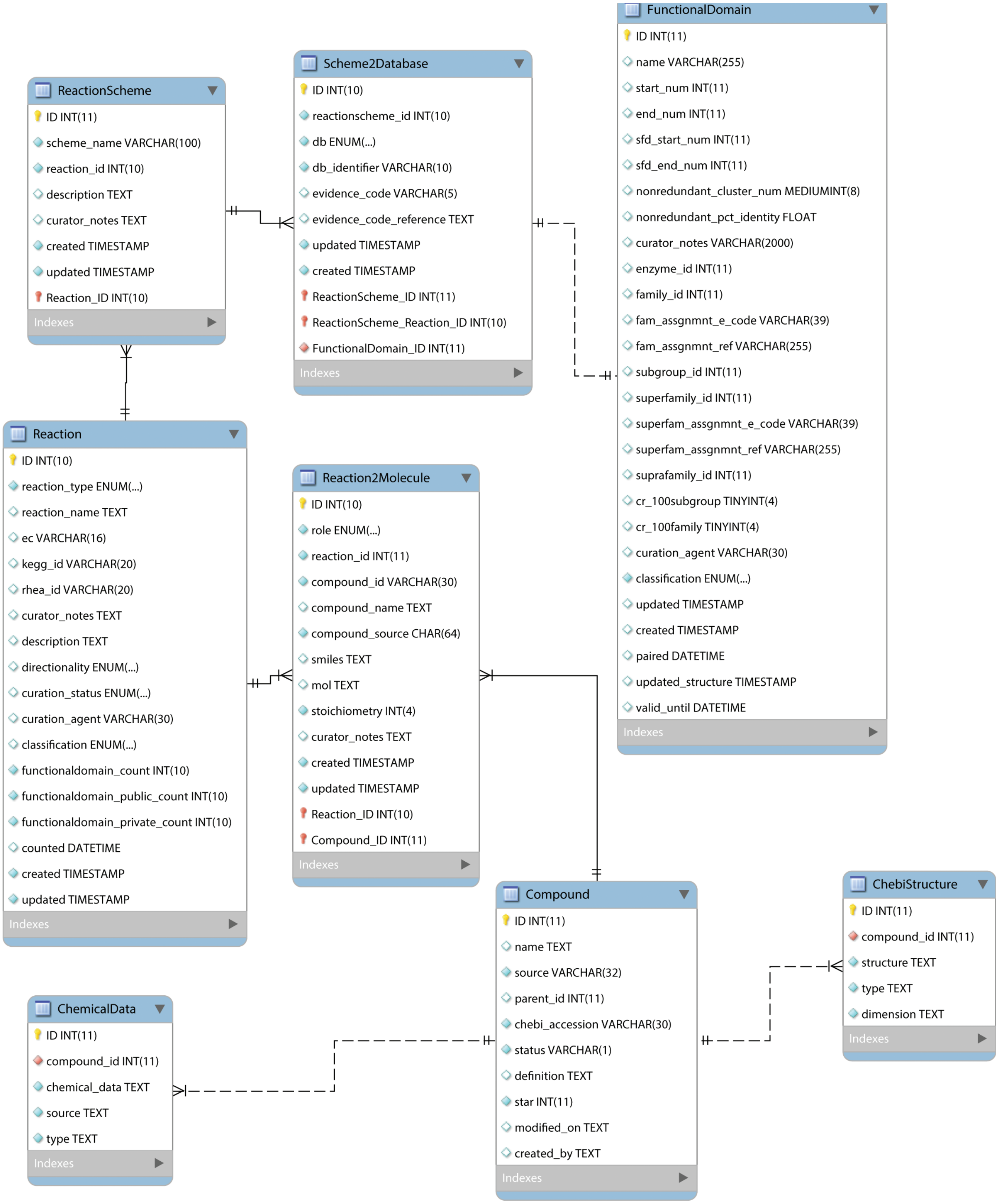

Fig S2. Full schema for reaction representation. The schema contains five tables unique to the MEERCat strategy (Reaction, ReactionScheme, Scheme2DB, Reaction2Molecule, FunctionalDomain) and three tables from ChEBI (Compound, ChemicalData & ChebiStructure).

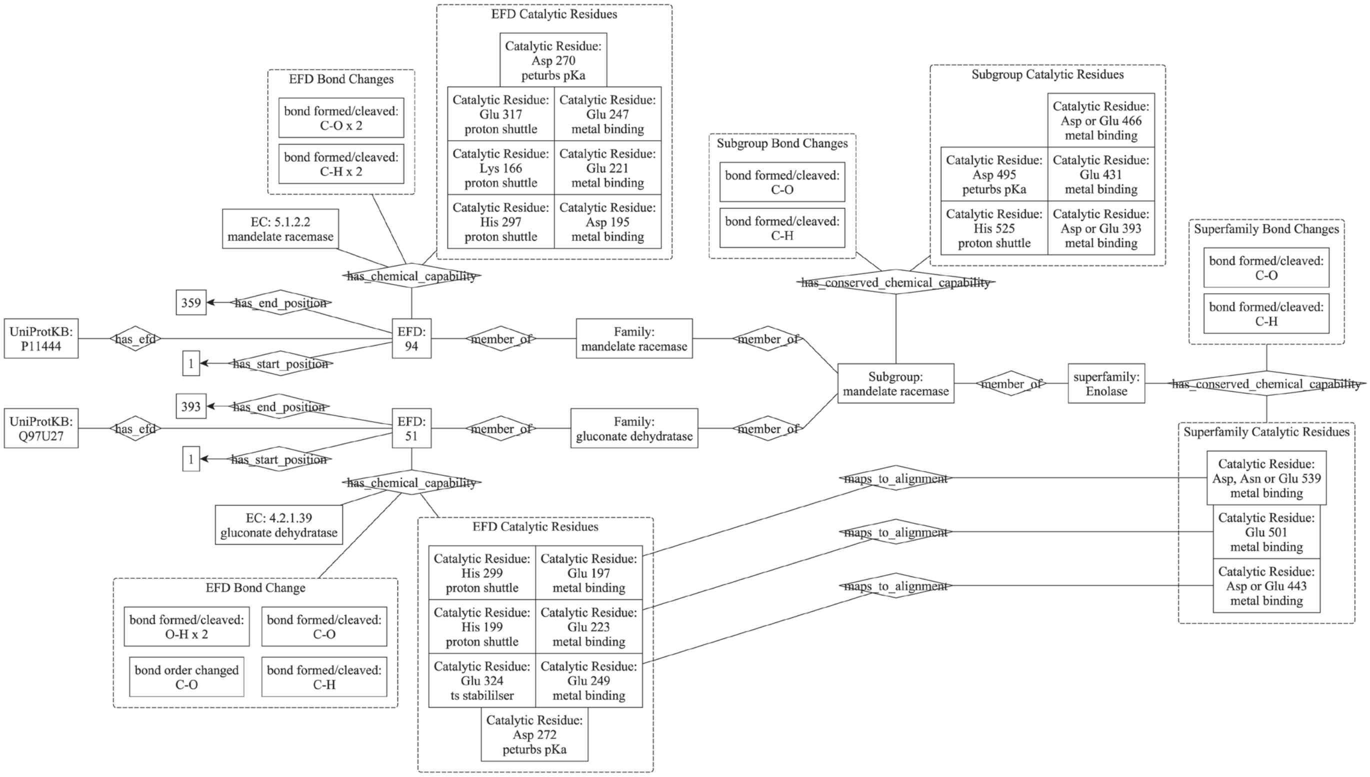

Figure S3. Detailed annotation of the chemical components associated with two members of the Enolase Superfamily. The conserved chemical components are inherited upward in the hierarchy so that they are linked for the subgroup and superfamily level annotations as well as for these specific family enzymes. The annotation at the family level has been omitted in this figure for simplicity, but is identical to that shown for the EFD (although the residue position numbers differ). The UniProtKB proteins P11444 (EFD 94, mandelate racemase) and Q97U27 (EFD 51, gluconate dehydratase) annotated in this figure are also used as examples in Figure S1.

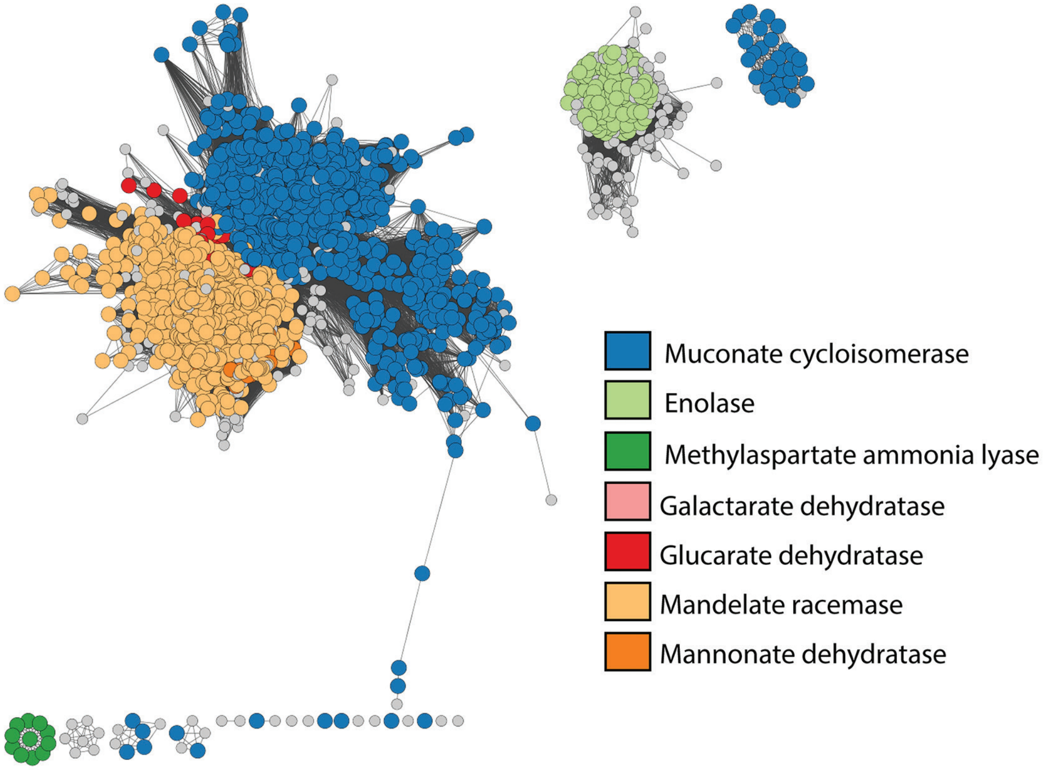

Figure S4. Representative sequence similarity network of the enolase superfamily. Enolase superfamily sequences were gathered from the SFLD in November 2017. Networks were generated from all-by-all pairwise comparisons of the 48,850 sequences using the BLAST algorithm. CD-Hit (3) was used to cluster the sequence set at 50% pairwise identity, producing 1,828 representative nodes, each representing from 1 to 8,914 sequences. Networks were generated using algorithms inspired by the Pythoscape software (4) tailored for use with available hardware. Edges are drawn between two nodes only if the mean similarity between the sequences in each node is at least as significant as the E-value of 1x10-20 (used as a score) (5) chosen to illustrate similarity relationships for the network. The nodes are visualized using Cytoscape (6) and arranged using the Organic layout.

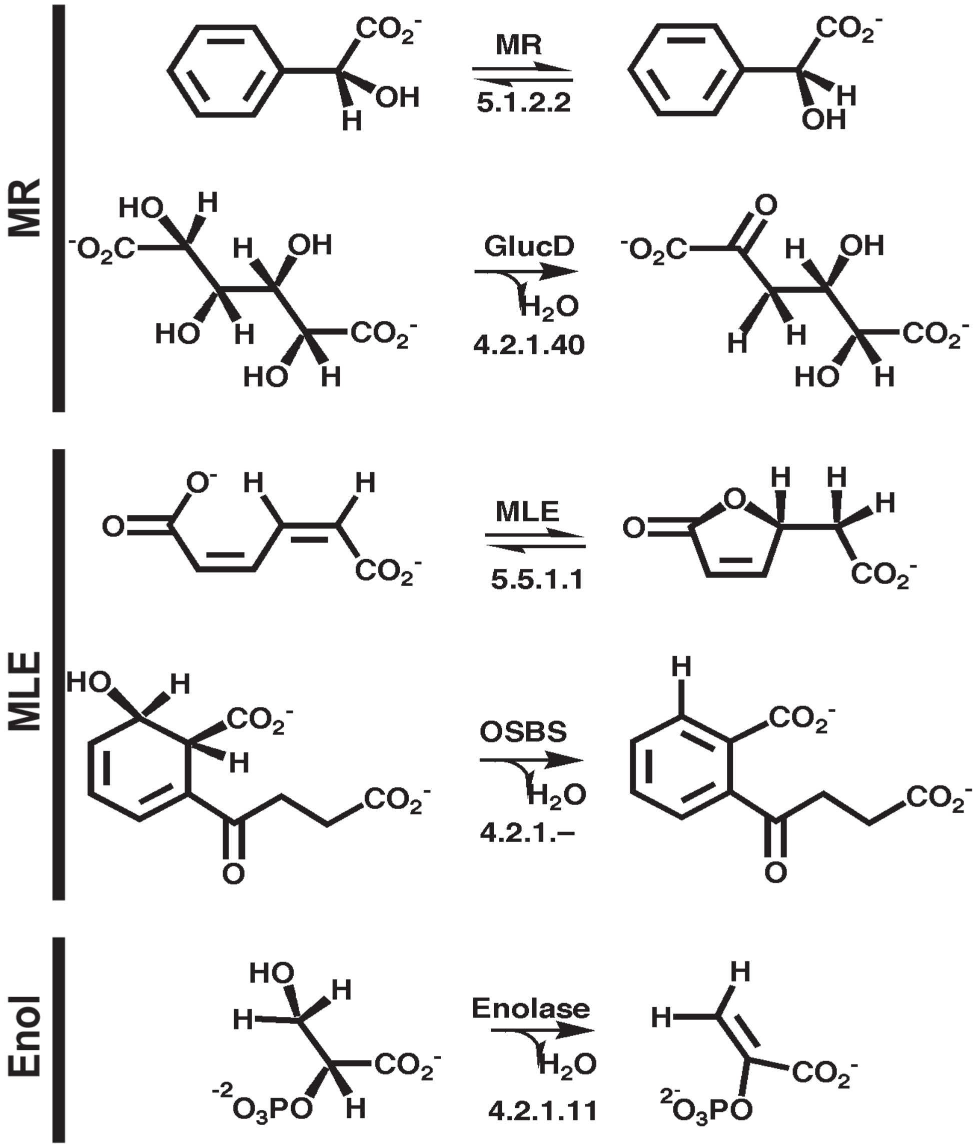

Figure S5. A sampling of chemical reactions of the MR, MLE, and Enol subgroups. Representative reactions and their associated EC numbers illustrate the range of EC numbers represented in the known reactions of the Enolase superfamily.

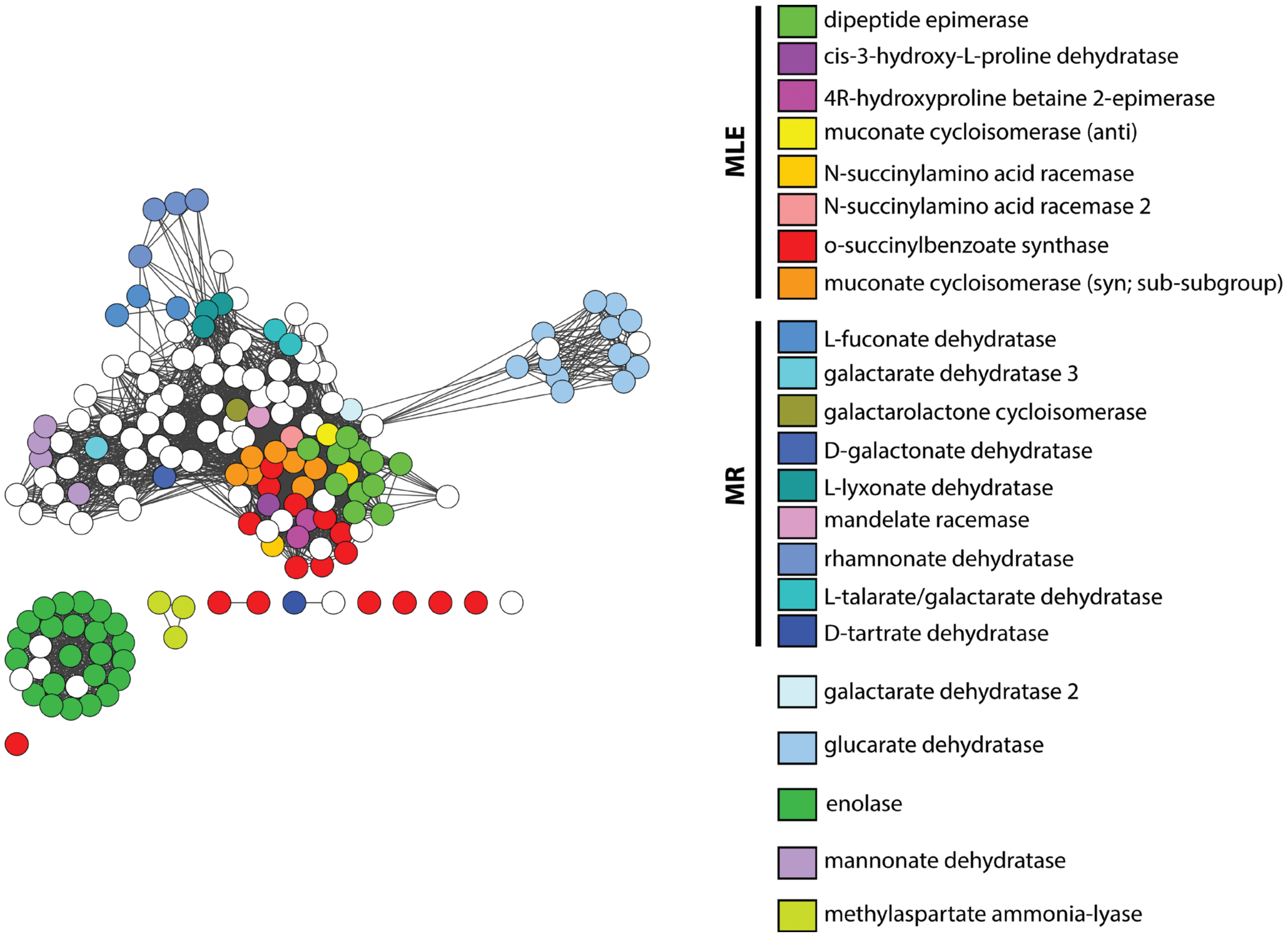

Figure S6. Enolase family mappings to structure similarity network. The same structure similarity network as in Figure 4A colored by families with structures available in the SFLD. Labeled black lines group families of the MLE and MR subgroups respectively. The five separate subgroups listed at the bottom of the key are currently thought to contain only one biochemically characterized monofunctional family.

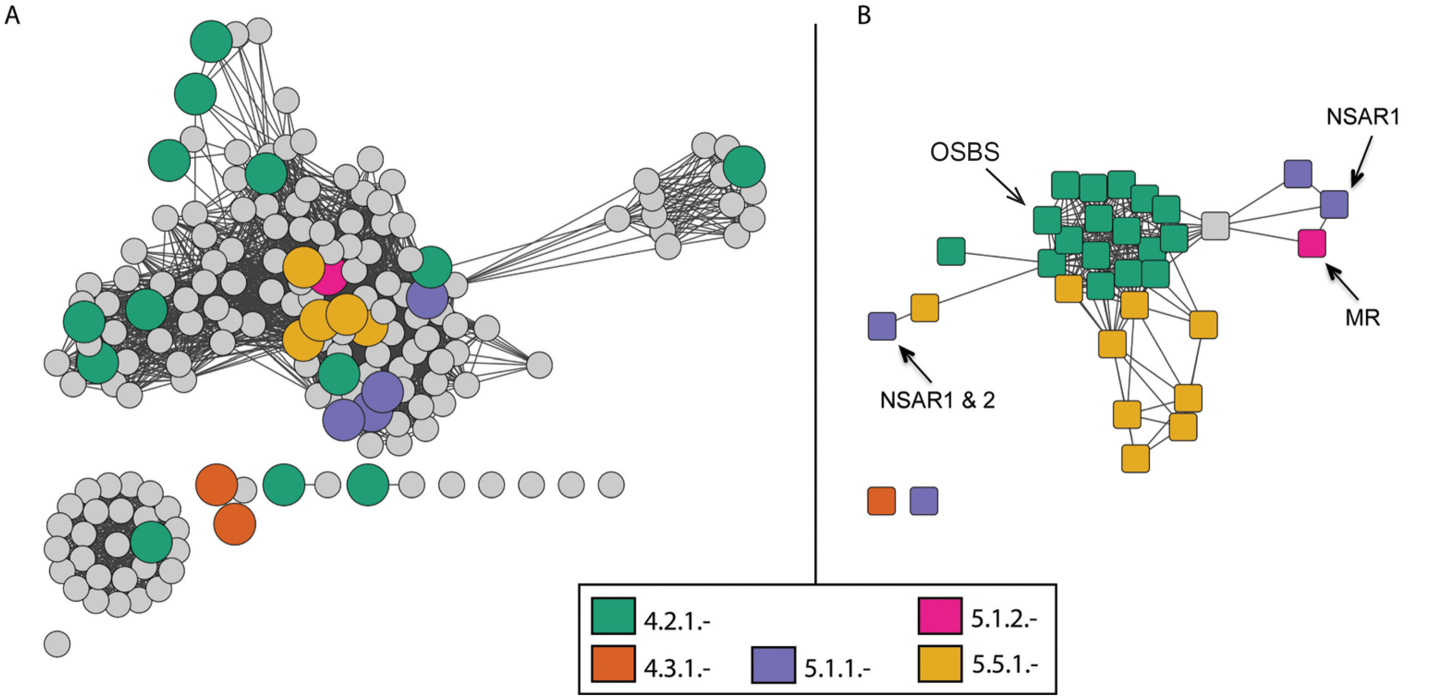

Figure S7. Structure- and chemical-similarity networks colored by EC chemical classification. A) Structure similarity network as in Figure 4A except with enlarged nodes colored by the first three digits of their EC numbers (designating their overall reactions but not their substrate specificities). Smaller gray nodes represent reactions that have not yet been assigned an EC number by the Enzyme Nomenclature Commission. B) Reaction similarity network as in Figure 4B.

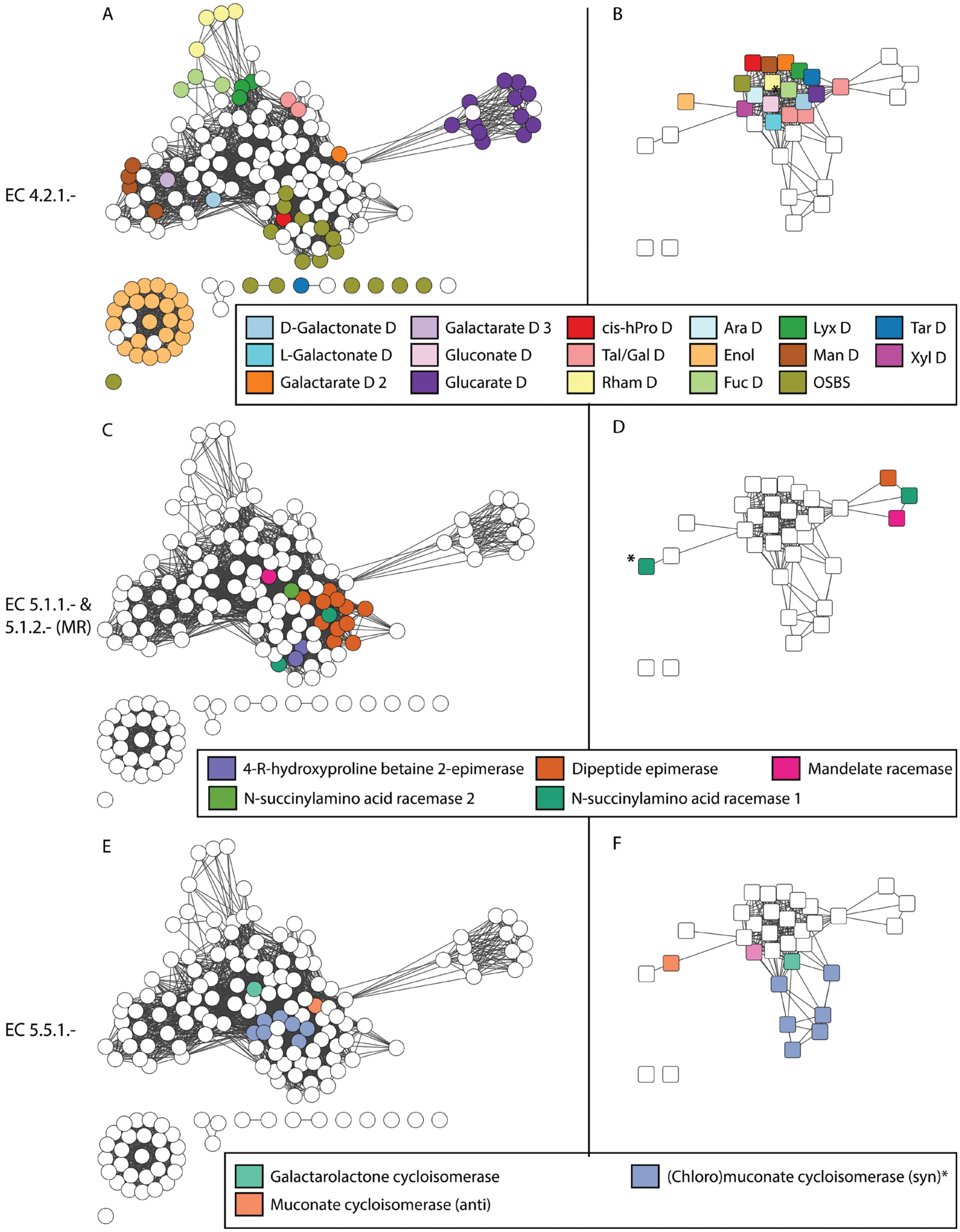

Figure S8: Mappings of families to structure and reaction networks to reaction sets classified by their first three EC numbers. Each pair of structure and reaction networks, AB, CD, EF, respectively, is associated with a group of EC numbers designating those families' overall reaction classifications. Set CD designates families of EC 5.1.1 class except for mandelate racemase, for which the overall reaction is classified to EC 5.1.2. The green node named as N-succinylamino acid racemase 2 is associated only with panel C. The reaction indicated by the asterisk in panel D is found in both the NSAR1 and NSAR2 families (both shown in turquoise in panel D). Details for classification assignments are available from the SFLD archive.

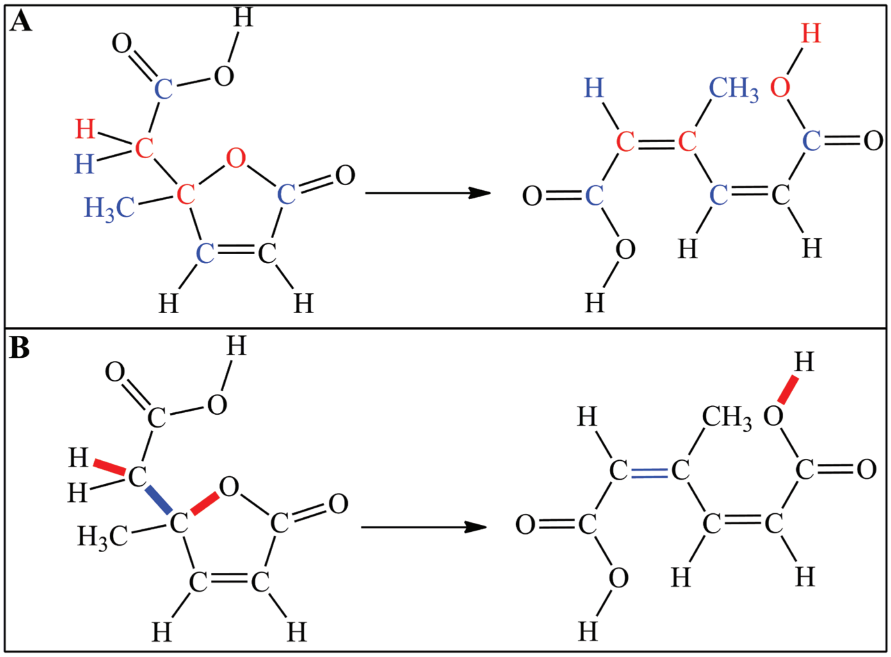

Figure S9. Muconate cycloisomerase reaction shown post atom-atom mapping. A) Reaction center highlighted in which red atoms are those at which a bond change occurs and blue atoms are the n+1 reaction center. B) Bond changes highlighted in which red bonds are those formed/ cleaved and blue bonds are those that are changed in order during the course of the reaction.

S1 Table. EC classification for Enolase superfamily members in the SFLD.

Subgroup (Level 1)*	Subgroup (Level 2)	Family	EC
enolase		enolase	4.2.1.11
galactarate dehydratase		galactarate dehydratase 2	4.2.1.-
glucarate dehydratase		glucarate dehydratase	4.2.1.40
**mandelate racemase**		3,6-anhydro-alpha-L-galactonate cycloisomerase	5.5.1.25
		D-arabinonate dehydratase	4.2.1.5
		D-galactonate dehydratase	4.2.1.6
		D-tartrate dehydratase	4.2.1.81
		galactarate dehydratase 3	4.2.1.-
		galactarolactone cycloisomerase	5.5.1.-
		gluconate dehydratase	4.2.1.39
		L-fuconate dehydratase	4.2.1.68
		L-galactonate dehydratase	4.2.1.146
		L-lyxonate dehydratase	4.2.1.-
		L-talarate/galactarate dehydratase	4.2.1.42, 4.2.1.156, 5.1.-.-
		mandelate racemase	5.1.2.2
		rhamnonate dehydratase	4.2.1.90
		xylonate dehydratase 1	4.2.1.82
		xylonate dehydratase 2	4.2.1.82
mannonate dehydratase		mannonate dehydratase	4.2.1.8
methylaspartate ammonia-lyase		methylaspartate ammonia-lyase	4.3.1.2
**muconate cycloisomerase**		4R-hydroxyproline betaine 2-epimerase	5.1.1.22
		cis-3-hydroxy-L-proline dehydratase	4.2.1.-
		dipeptide epimerase	5.1.1.20
		muconate cycloisomerase (anti)	5.5.1.1
		N-succinylamino acid racemase 1	5.1.-.-
		N-succinylamino acid racemase 2	5.1.-.-
		o-succinylbenzoate synthase	4.2.1.113
	muconate cycloisomerase (syn)		5.5.1.-

* Subgroups containing ≥ 2 families are shown in bold typeface.

References

1. Finn, R.D., Attwood, T.K., Babbitt, P.C., et al. (2017) InterPro in 2017-beyond protein family and domain annotations. Nucleic Acids Res, 45, D190-D199.
2. Gulick, A.M. (2009) Conformational dynamics in the Acyl-CoA synthetases, adenylation domains of non-ribosomal peptide synthetases, and firefly luciferase. ACS chemical biology, 4, 811-827.
3. Fu, L., Niu, B., Zhu, Z., et al. (2012) CD-HIT: accelerated for clustering the next-generation sequencing data. Bioinformatics, 28, 3150-3152.
4. Barber, A.E., 2nd, Babbitt, P.C. (2012) Pythoscape: A framework for generation of large protein similarity networks. Bioinformatics.
5. Atkinson, H.J., Morris, J.H., Ferrin, T.E., et al. (2009) Using sequence similarity networks for visualization of relationships across diverse protein superfamilies. PLoS One, 4, e4345.
6. Smoot, M.E., Ono, K., Ruscheinski, J., et al. (2011) Cytoscape 2.8: new features for data integration and network visualization. Bioinformatics, 27, 431-432.
